# Epigenetic-Mediated Downregulation of Zinc Finger Protein 671 (*ZNF671*) Predicts Poor Prognosis in Multiple Solid Tumors

**DOI:** 10.3389/fonc.2019.00342

**Published:** 2019-05-07

**Authors:** Jian Zhang, Ziqi Zheng, Jieling Zheng, Tao Xie, Yunhong Tian, Rong Li, Baiyao Wang, Jie Lin, Anan Xu, Xiaoting Huang, Yawei Yuan

**Affiliations:** ^1^Department of Radiation Oncology, Affiliated Cancer Hospital and Institute of Guangzhou Medical University, Guangzhou, China; ^2^State Key Laboratory of Respiratory Diseases, Guangzhou Institute of Respiratory Disease, Affiliated Cancer Hospital and Institute of Guangzhou Medical University, Guangzhou, China; ^3^State Key Laboratory of Oncology in South China, Collaborative Innovation Center of Cancer Medicine, Sun Yat-sen University Cancer Center, Guangzhou, China; ^4^Department of Radiation Oncology, Nanfang Hospital, Southern Medical University, Guangzhou, China

**Keywords:** epigenetic, *ZNF671*, prognosis, solid tumor, data mining

## Abstract

Zinc finger protein 671 (*ZNF671*) is a member of the largest transcription factor family in the human genome. However, the methylation status, expression, and prognostic role of *ZNF671* in solid tumors remain unclear. The aim of this study was to explore the relationship between *ZNF671* and the prognosis of patients with solid tumors. We performed a pan-cancer analysis of the methylation status and mRNA and protein expression of *ZNF671* using The Cancer Genome Atlas (TCGA) database and the Human Protein Atlas. We further evaluated the prognostic value of *ZNF671* expression among numerous cancer types using the “Kaplan–Meier plotter” (KM plotter) database. We found that downregulation of *ZNF671* is associated with hypermethylation of its promoter. Survival analysis established that the downregulation of *ZNF671* predicts poor prognosis in breast invasive carcinoma (BRCA), cervical squamous cell carcinoma and endocervical adenocarcinoma (CESC), head and neck squamous cell carcinoma (HNSC), kidney renal papillary cell carcinoma (KIRP), lung adenocarcinoma (LUAD), pancreatic adenocarcinoma (PAAD), and uterine corpus endometrial carcinoma (UCEC) solid tumors. CCK-8 and Transwell functional assays showed that ZNF671 could inhibit tumor cell proliferation, migration, and invasion. These results indicate that *ZNF671* is an excellent predictive factor for BRCA, CESC, HNSC, KIRP, LUAD, PAAD, SARC, and UCEC solid tumors and may play crucial roles in the development and progression of these tumors.

## Introduction

Epigenetic regulation is essential for the normal development and maintenance of gene expression in mammals. Many biological processes are involved in DNA methylation, histone modification, and nucleosome remodeling (1). Disruption of epigenetic processes can lead to activation of oncogenes and/or the inactivation of tumor suppressor pathways, and the accumulation of epigenetic changes in the molecular landscape is a hallmark of cancer (2). DNA methylation is an early event in tumorigenesis and plays a major role in tumor initiation and progression (3). Therefore, it is important to identify reliable prognostic and predictive biomarkers in solid tumors that may help in early diagnosis and treatment strategies.

Zinc finger (ZF) protein 671 (*ZNF671*) is a member of the KRAB-ZF (KRAB-ZFP) family of mammalian transcriptional repressors (4–6). Through recruitment of KRAB-associated protein-1 and other co-repressors, KRAB-ZFPs can regulate cell differentiation, proliferation, apoptosis, tumor suppression, and neoplastic transformation (7–11). The Illumina Human Methylation 450K Beadchip analysis and genome-wide methylation profiles identified *ZNF671* as a gene with significantly higher methylation compared to other genes (12, 13). However, the methylation level and prognostic role of *ZNF671* in other tumors are still not clear.

In this study, we systematically analyzed the methylation level and expression of *ZNF671* and explored its correlation with solid tumors by analyzing The Cancer Genome Atlas (TCGA) database and the Human Protein Atlas (HPA). We evaluated the prognostic role of *ZNF671* in 18 solid tumors using the Kaplan–Meier (KM) plotter database. Our results provide important insights into *ZNF671* hypermethylation as a promising biomarker for eight solid tumors and thereby provide novel perspectives for the treatment of tumors.

## Materials and Methods

### TCGA Dataset Analysis

Gene methylation and expression data were downloaded from TCGA (http://cancergenome.nih.gov) and were interpreted, normalized, and log2 scaled using the online analysis tool. This study was approved by the Institutional Ethical Review Boards of Guangzhou Medical University Cancer Center.

### Immunohistochemistry Analysis

The HPA database (https://www.proteinatlas.org/) was used to perform a pan-cancer analysis of ZNF671 protein expression (14). ZNF671 was entered in the database (https://www.proteinatlas.org/) to acquire ZNF671 expression for individual tumors of each cancer type (Scar bar = 200μm). All IHC images have been manually annotated by certified pathologists.

### Prognostic Analysis

An online KM plotter database was used to assess the correlation of intra-individual *ZNF671* mRNA expression with progression-free survival (15). *ZNF671* was entered in the database (http://kmplot.com/analysis/) to acquire pan-cancer KM survival plots. Hazard ratio (HR), 95% confidence intervals, and log-rank *P* were determined and presented on the main plots.

### Western Blot Analysis

After cells were transfected with the pSin-EF2-puro-*ZNF671*-HA or pSin-EF2-puro-vector plasmids (Land Hua Gene Biosciences, Guangzhou, China) for 48 h, Radio-Immunoprecipitation Assay (RIPA) lysis buffer (Beyotime, Shanghai, China) was used to isolate proteins. Proteins were separated by Sodium dodecyl sulfate (SDS)-polyacrylamide gel electrophoresis (SDS-PAGE, Beyotime), transferred onto polyvinylidene fluoride (PVDF) membranes (Millipore, Billerica, MA, USA), and incubated with primary anti-ZNF671 (1:500; Proteintech, Chicago, IL, USA) and anti-Glyceraldehyde-3-phosphate dehydrogenase (GAPDH) (1:1,000, Proteintech, Chicago, IL, USA).

### CCK-8 and Transwell Assays

For the CCK-8 assay, cells (1 × 10^3^) were seeded into 96-well plates, incubated for 0–4 days, stained with CCK-8 (Dojindo, Tokyo, Japan), and absorbance values were determined at 450 nm using a spectrophotometer. Transwell migration and invasion assays were carried out using Transwell chambers (8 μm; Corning, Tewksbury, MA, USA) without or with Matrigel (BD Biosciences, San Jose, CA, USA). Cells were added to the upper chamber in serum-free medium, and the lower chamber contained culture medium with 20% FBS. The cells were incubated for 12 or 24 h at 37°C in 5% CO_2_ and were then fixed and stained. Cells on the undersides of the filters were observed and counted under i200 magnification.

### Statistical Analysis

Statistical analysis was performed using SPSS version 17.0 (SPSS Inc., Chicago, IL, USA). Differences between two groups were analyzed using the two-tailed unpaired Student's *t*-test; *P* < 0.05 was considered statistically significant.

## Results

### *ZNF671* Promoter Is Hypermethylated in Several Solid Tumor Types

**Figure 1:**
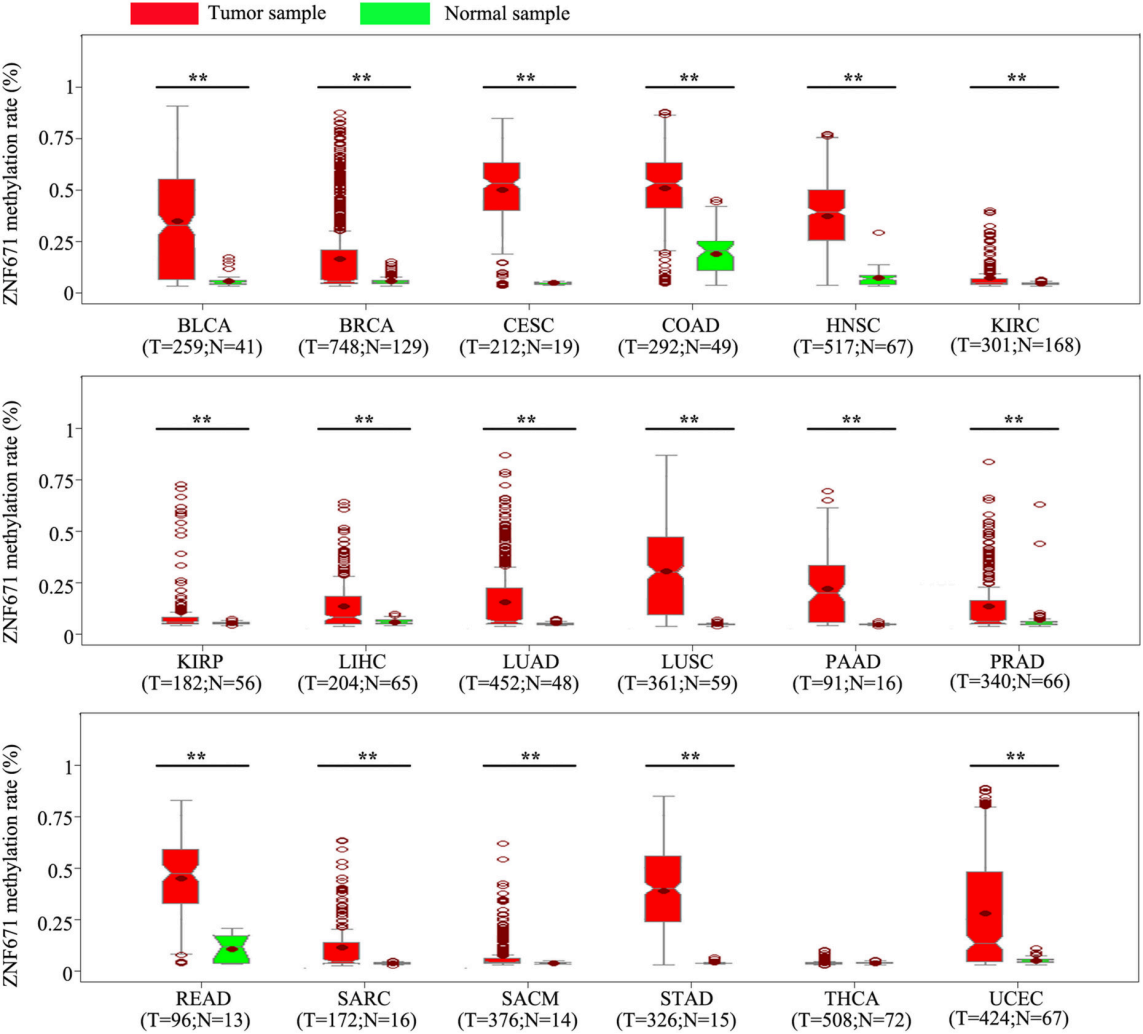
*ZNF671* is hypermethylated in solid tumors. *ZNF671* methylation analysis from The Cancer Genome Atlas. BLCA, bladder urothelial carcinoma; BRCA, breast invasive carcinoma; CESC, cervical squamous cell carcinoma and endocervical adenocarcinoma; COAD, colon adenocarcinoma; HNSC, head and neck squamous cell carcinoma; KIRC, kidney renal clear cell carcinoma; KIRP, kidney renal papillary cell carcinoma; LIHC, liver hepatocellular carcinoma; LUAD, lung adenocarcinoma; LUSC, lung squamous cell carcinoma; PAAD, pancreatic adenocarcinoma; PRAD, prostate adenocarcinoma; READ, rectum adenocarcinoma; SARC, sarcoma; SKCM, skin cutaneous melanoma; STAD, stomach adenocarcinoma; THCA, thyroid carcinoma; UCEC, uterine corpus endometrial carcinoma.

To determine the methylation level of *ZNF671* across cancer types, we analyzed human pan-cancer methylation data in the TCGA database. Among the 18 cancers examined, the promoter of *ZNF671* was hypermethylated in bladder urothelial carcinoma (BLCA), breast invasive carcinoma (BRCA), cervical squamous cell carcinoma and endocervical adenocarcinoma (CESC), colon adenocarcinoma (COAD), head and neck squamous cell carcinoma (HNSC), kidney renal clear cell carcinoma (KIRC), kidney renal papillary cell carcinoma (KIRP), liver hepatocellular carcinoma (LIHC), lung adenocarcinoma (LUAD), lung squamous cell carcinoma (LUSC), pancreatic adenocarcinoma (PAAD), prostate adenocarcinoma (PRAD), rectum adenocarcinoma (READ), sarcoma (SARC), skin cutaneous melanoma (SKCM), stomach adenocarcinoma (STAD), and uterine corpus endometrial carcinoma (UCEC) (Figure 1, ^**^P < 0.01). For thyroid carcinoma (THCA), the methylation level between tumor tissues and normal tissues did not differ.

### *ZNF671* mRNA Expression Is Downregulated in Several Solid Tumor Types

**Figure 2:**
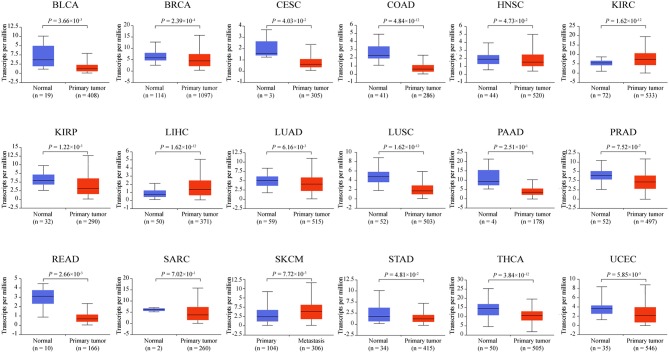
*ZNF671* is downregulated in primary solid tumors. *ZNF671* mRNA expression analysis from The Cancer Genome Atlas. BLCA, bladder urothelial carcinoma; BRCA, breast invasive carcinoma; CESC, cervical squamous cell carcinoma and endocervical adenocarcinoma; COAD, colon adenocarcinoma; HNSC, head and neck squamous cell carcinoma; KIRC, kidney renal clear cell carcinoma; KIRP, kidney renal papillary cell carcinoma; LIHC, liver hepatocellular carcinoma; LUAD, lung adenocarcinoma; LUSC, lung squamous cell carcinoma; PAAD, pancreatic adenocarcinoma; PRAD, prostate adenocarcinoma; READ, rectum adenocarcinoma; SARC, sarcoma; SKCM, skin cutaneous melanoma; STAD, stomach adenocarcinoma; THCA, thyroid carcinoma; UCEC, uterine corpus endometrial carcinoma.

**Figure 3:**
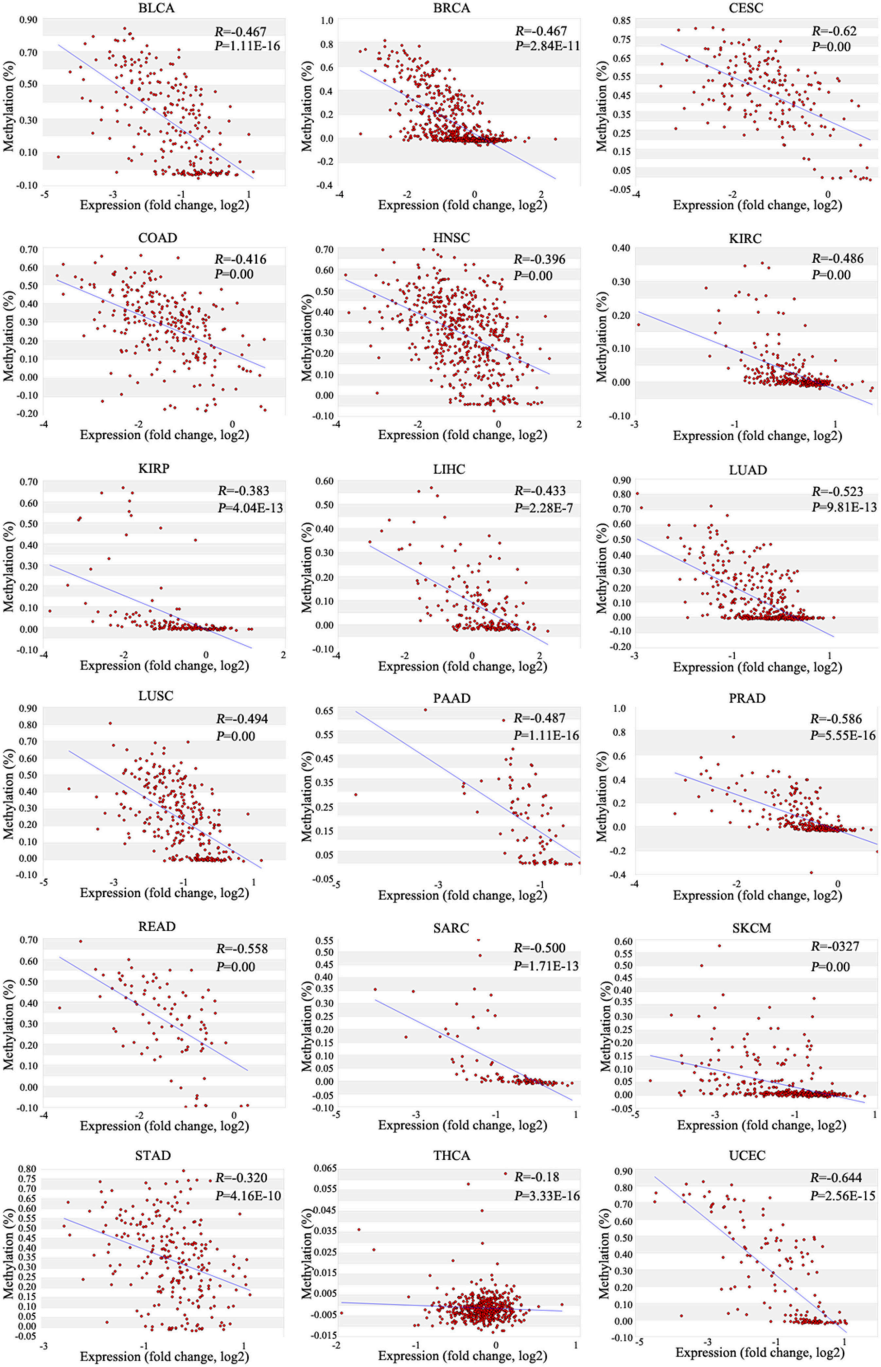
Correlations between *ZNF671* promoter methylation and mRNA expression in primary solid tumors. Analysis of data from The Cancer Genome Atlas. BLCA, bladder urothelial carcinoma; BRCA, breast invasive carcinoma; CESC, cervical squamous cell carcinoma and endocervical adenocarcinoma; COAD, colon adenocarcinoma; HNSC, head and neck squamous cell carcinoma; KIRC, kidney renal clear cell carcinoma; KIRP, kidney renal papillary cell carcinoma; LIHC, liver hepatocellular carcinoma; LUAD, lung adenocarcinoma; LUSC, lung squamous cell carcinoma; PAAD, pancreatic adenocarcinoma; PRAD, prostate adenocarcinoma; READ, rectum adenocarcinoma; SARC, sarcoma; SKCM, skin cutaneous melanoma; STAD, stomach adenocarcinoma; THCA, thyroid carcinoma; UCEC, uterine corpus endometrial carcinoma.

To determine the mRNA expression level of *ZNF671* in different cancers, we further used the TCGA database to validate the mRNA expression of *ZNF671* and found that *ZNF671* was downregulated in BLCA, BRCA, CESC, COAD, HNSC, KIRP, LUAD, LUSC, PAAD, PRAD, READ, SARC, STAD, THCA, and UCEC, but the mRNA expression of *ZNF671* was upregulated in KIRC, LIHC, and SKCM (Figure 2). Further correlation analysis found that the mRNA expression was negatively correlated with promoter methylation in the 18 cancers (Figure 3). These results indicate that the promoter hypermethylation of *ZNF671* mediates its downregulation in BLCA, BRCA, CESC, COAD, HNSC, KIRP, LUAD, LUSC, PAAD, PRAD, READ, SARC, STAD, THCA, and UCEC cancers.

### ZNF671 Protein Expression Is Downregulated in Several Solid Tumor Types

**Figure 4:**
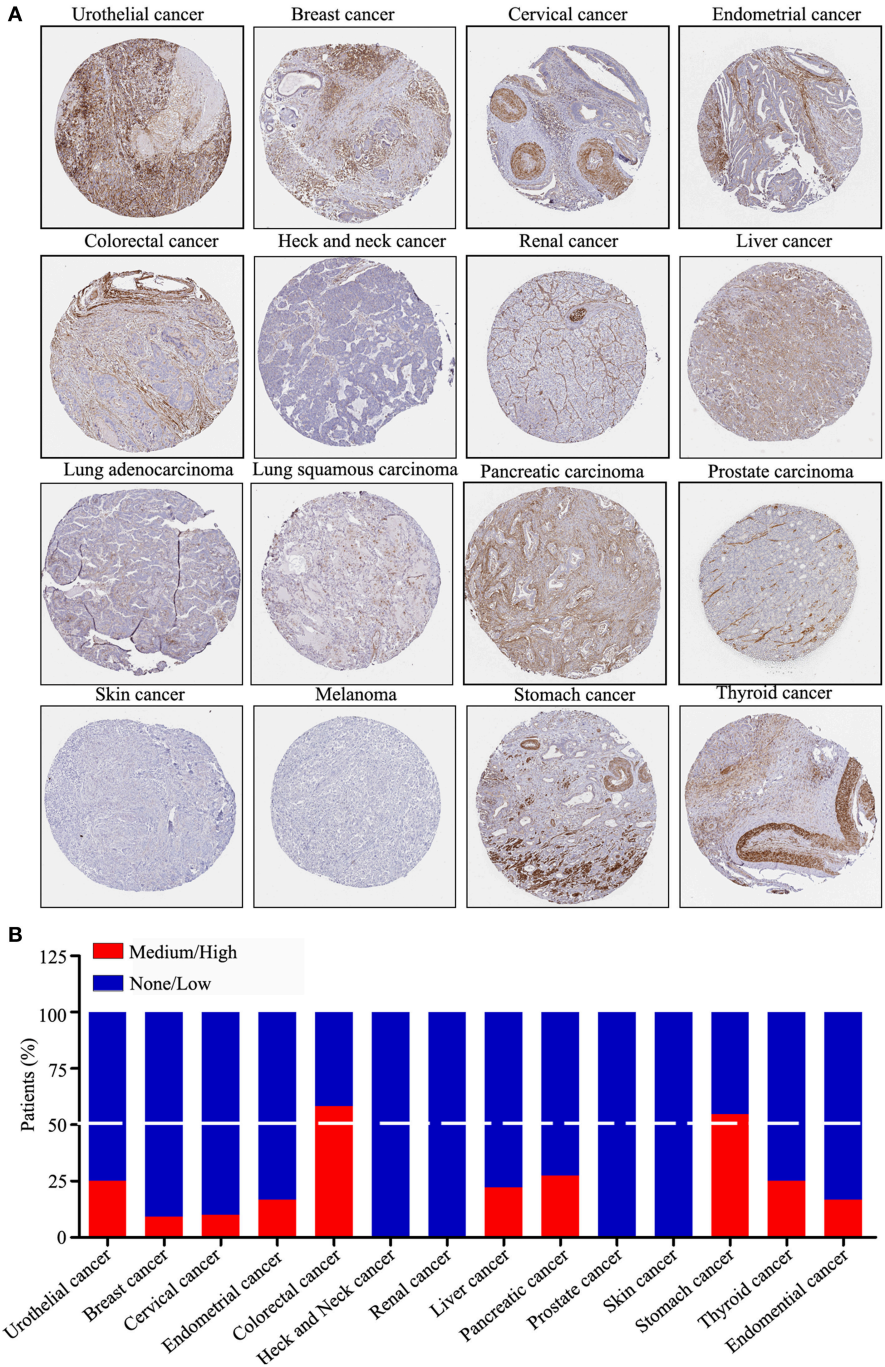
ZNF671 protein expression is downregulated in primary solid tumors. **(A)** Immunohistochemical stained tissues representing ZNF671 protein expression as shown in the Human Pathology Atlas database. **(B)** Protein expression levels across 14 cancer types analyzed by IHC in the Human Pathology Atlas database.

We performed a pan-cancer analysis of the protein expression of ZNF671 using the HPA, which presented the protein expression of ZNF671 in 17 different tumor types. As shown in Figure 4A, the protein expression level of ZNF671 was downregulated in most cancers. Statistical analysis indicated that ZNF671 expression was low and/or absent in urothelial (75%), breast (90.9%), cervical (90%), endometrial (83.3%), head and neck (100%), renal (100%), liver (77.8%), lung (100%), pancreatic (72.7%), prostate (100%), skin (100%), and thyroid (75%) cancers. ZNF671 protein expression was low and/or absent in only 41.7 and 45.4% of colorectal and stomach cancers, respectively (Figure 4B), and the protein expression of ZNF671 in sarcoma was unclear. These results indicate that ZNF671 protein expression is downregulated in urothelial, breast, cervical, endometrial, heck and neck, renal, liver, lung, pancreatic, prostate, skin, and thyroid cancers and that ZNF671 may play a tumor suppressor role in cancer progression.

### *ZNF671* Is a Prognostic Biomarker in Several Solid Tumor Types

**Figure 5:**
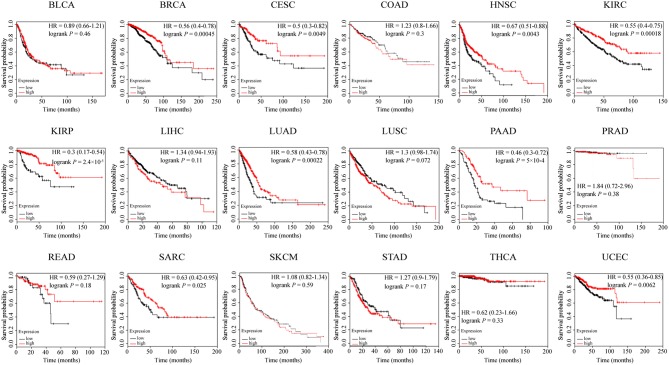
The prognostic effect of *ZNF671* expression in solid tumors from KM plotter. Survival curves are plotted for all patients. BLCA, bladder urothelial carcinoma; BRCA, breast invasive carcinoma; CESC, cervical squamous cell carcinoma and endocervical adenocarcinoma; COAD, colon adenocarcinoma; HNSC, head and neck squamous cell carcinoma; KIRC, kidney renal clear cell carcinoma; KIRP, kidney renal papillary cell carcinoma; LIHC, liver hepatocellular carcinoma; LUAD, lung adenocarcinoma; LUSC, lung squamous cell carcinoma; PAAD, pancreatic adenocarcinoma; PRAD, prostate adenocarcinoma; READ, rectum adenocarcinoma; SARC, sarcoma; SKCM, skin cutaneous melanoma; STAD, stomach adenocarcinoma; THCA, thyroid carcinoma; UCEC, uterine corpus endometrial carcinoma.

To further assess the prognostic role of *ZNF671* in different cancers, we explored the relationship between mRNA expression of *ZNF671* and patient survival using an online tool (http://kmplot.com). High *ZNF671* mRNA expression was found to be significantly correlated with increased probability of survival for BRCA (HR = 0.56; *P* = 0.00045), CESC (HR = 0.5; *P* = 0.0049), HNSC (HR = 0.67; *P* = 0.0043), KIRC (HR = 0.55; *P* = 0.00018), KIRP (HR = 0.3; *P* = 2.4e-5), LUAD (HR = 0.58; *P* = 0.00022), PAAD (HR = 0.46; *P* = 5e-4), SARC (HR = 0.63; *P* = 0.025), and UCEC (HR = 0.55; *P* = 0.0062), but not in BLCA (HR = 0.89; *P* = 0.46), COAD (HR = 1.23; *P* = 0.3), LIHC (HR = 1.34; *P* = 0.11), LUSC (HR = 1.3; *P* = 0.072), PRAD (HR = 1.84; *P* = 0.38), READ (HR = 0.59; *P* = 0.18), SKCM (HR = 1.08; *P* = 0.025), STAD (HR = 1.27; *P* = 0.17), and THCA (HR = 0.62; *P* = 0.33; Figure 5).

The relationship between *ZNF671* methylation, mRNA and protein expression, and prognosis among different cancer types is summarized in Table 1. Overall, the results indicate that epigenetic-mediated downregulation of *ZNF671* predicts poor survival in BRCA, CESC, HNSC, KIRP, LUAD, PAAD, UCEC, and SARC and that *ZNF671* may play a tumor suppressor role in tumor progression, which is consistent with previous studies in HNSC, UCEC, and KIRP (11, 16, 17).

**Table 1:** Methylation status, gene expression, protein expression (IHC), correlation analysis and prognostic predictive value of *ZNF671* in 18 solid tumors.

**Tumor**	**Methylation**	**Expression**	**IHC**	**Correlation**	**Prognosis**
BLCA	√	√	√	√	×
BRCA	√	√	√	√	√
CESC	√	√	√	√	√
COAD	√	√	×	√	×
HNSC	√	√	√	√	√
KIRC	√	×	√	√	√
KIRP	√	√	√	√	√
LIHC	√	×	√	√	×
LUAD	√	√	√	√	√
LUSC	√	√	√	√	×
PAAD	√	√	√	√	√
PRAD	√	√	√	√	×
READ	√	√	√	√	×
SARC	√	√	NA	√	√
SKCM	√	×	√	√	×
STAD	√	√	×	√	×
THCA	×	√	√	×	×
UCEC	√	√	√	√	√

*√ means can search on database; × means can't search on database; NA means “not known” on database*.

### *ZNF671* Inhibits Tumor Cell Proliferation, Migration, and Invasion

**Figure 6:**
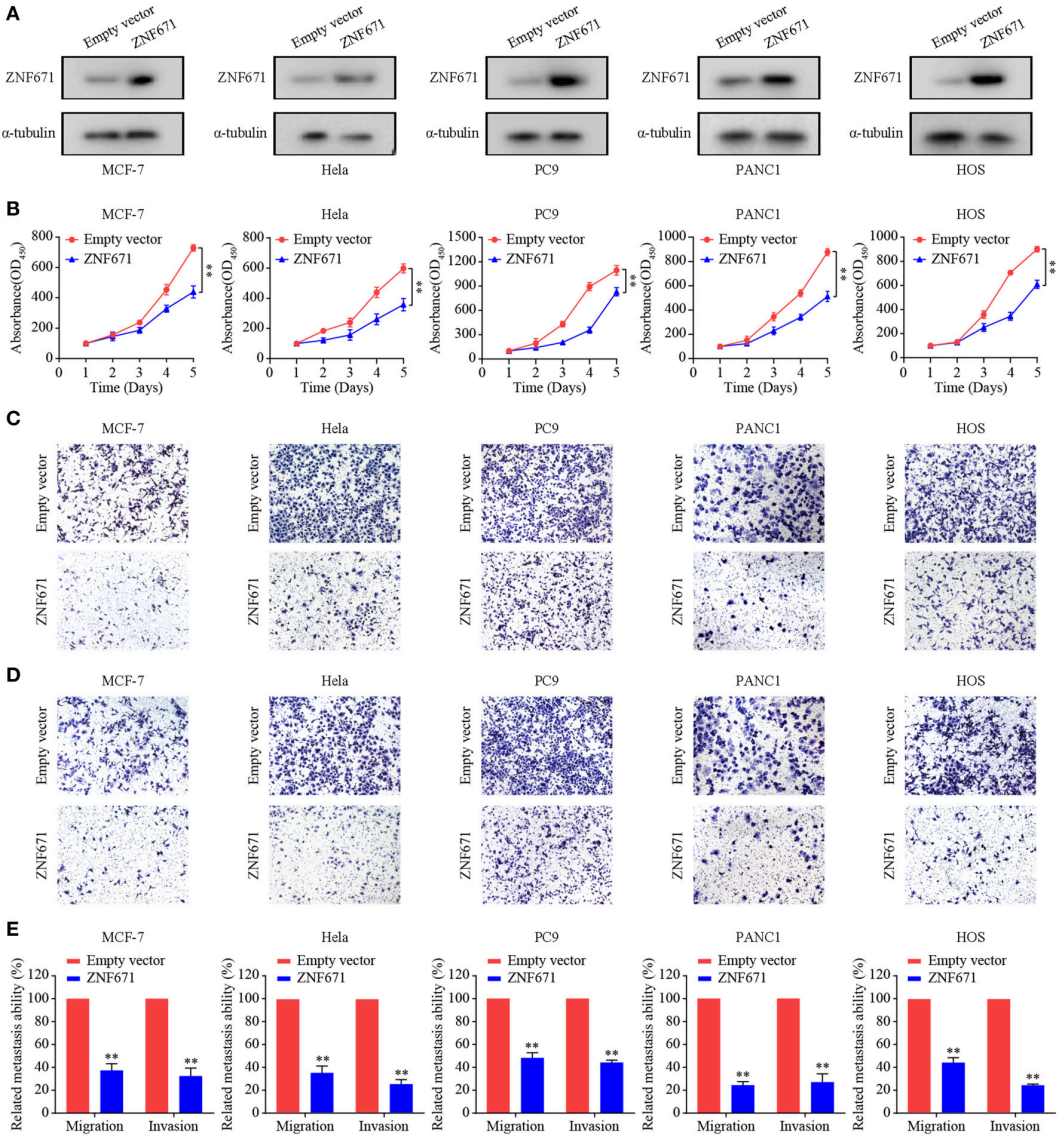
Effects of ZNF671 overexpression on cell proliferation, migration, and invasion *in vitro*. **(A)** Representative Western blot analysis of ZNF671 overexpression in MCF7, Hela, PC9, PANC1, and HOS cell lines. **(B)** The CCK-8 assay showed overexpression of ZNF671 reduced the viability of MCF7, Hela, PC9, PANC1, and HOS cells. **(C,D)** Representative images of the effects of ZNF671 overexpression on the migratory and invasive abilities of cells as determined by the Transwell migration **(C)** and invasion **(D)** assays. **(E)** Quantification of the effects of ZNF671 overexpression on migratory and invasive abilities. All of the experiments were performed at least three times. Data presented are the mean ± SD; **P < 0.01. All of the experiments were performed at leas*t*-test.

To assess the effects of ZNF671 in BRCA, CESC, LUAD, PAAD, and SARC, we performed CCK-8 and Transwell assays using MCF7, Hela, PC9, PANC1, and HOS cells transfected with *ZNF671* or vector. As shown in Figure 6A, Western blot analysis validated that ZNF671 protein level was obviously elevated after transfection of *ZNF671* plasmid in MCF7, Hela, PC9, PANC1, and HOS cells. Overexpression of ZNF671 suppressed the proliferation (Figure 6B) and migratory and invasive (Figures 6C–E) abilities of the tumor cells as determined by Transwell migration and invasion assays. These findings indicate that ZNF671 inhibits the proliferation and migratory and invasive abilities of BRCA, CESC, LUAD, PAAD, and SARC cells *in vitro*.

## Discussion

*ZNF671* belongs to the KRAB-ZF family of transcription factors, which contain C2H2-type ZFs and a Krüppel-associated box (KRAB) domain. KRAB-ZFPs are involved in regulating cell differentiation, proliferation, apoptosis, tumor suppression, and immune control (7, 9, 11, 18, 19). Our previous studies demonstrated that *ZNF671* is a tumor suppressor that is epigenetically silenced by DNA methylation in nasopharyngeal carcinoma, but there is limited information regarding the role of *ZNF671* methylation among different solid cancer types. The present study found that downregulation of *ZNF671* was associated with its promoter hypermethylation among many different cancer types. Functional assays and prognostic analysis found that high *ZNF671* expression suppressed tumor cell proliferation, migration, and invasion, and downregulation of *ZNF671* was associated with poor clinical prognosis in BRCA, CESC, HNSC, KIRP, LUAD, PAAD, and UCEC. Thus, *ZNF671* represents a novel predictive marker for the prognosis of patients with BRCA, CESC, HNSC, KIRP, LUAD, PAAD, SARC, and UCEC.

In this study, we found that the *ZNF671* promoter is significantly methylated in 17 solid tumors, based on the TCGA datasets, which is consistent with studies in urothelial carcinoma, clear cell renal cell carcinomas, and cervical cancer (16, 17, 20). Next, we compared and analyzed the mRNA and protein expression levels of *ZNF671* in these solid tumors. Statistical analysis revealed that the expression of *ZNF671* was negatively correlated with its promoter hypermethylation in BLCA, BRCA, CESC, HNSC, KIRC, KIRP, LIHC, LUAD, LUSC, PAAD, READ, SARC, SKCM, and UCEC, suggesting a crucial role for *ZNF671* in the progression of these cancer types.

To further explore the potential prognostic value of *ZNF671* in BLCA, BRCA, CESC, HNSC, KIRC, KIRP, LIHC, LUAD, LUSC, PAAD, READ, SARC, SKCM, and UCEC, we used KM plotter analysis to evaluate the correlation between *ZNF671* expression and survival probability. KM plotter is used to analyze individual genes in different cancers (21). We found that low *ZNF671* expression may predict poorer survival in BRCA, CESC, HNSC, KIRP, LUAD, PAAD, SARC, and UCEC. CCK-8 and Transwell migration and invasion assays showed that ZNF671 could inhibit tumor cell proliferation, migration, and invasion. However, additional studies are still needed to explore the molecular mechanism underlying the effects of *ZNF671* expression in BRCA, CESC, HNSC, KIRP, LUAD, PAAD, SARC, and UCEC.

In conclusion, this is the first report to systematically evaluate the correlation between methylation of *ZNF671* and prognosis among 18 solid tumors. The prognostic signature observed in our study was linked to hypermethylation of *ZNF671*. Our findings reveal that *ZNF671* is hypermethylated in BRCA, CESC, HNSC, KIRP, LUAD, PAAD, SARC, and UCEC, and functional assays established that ZNF671 inhibits tumor cell proliferation, migration, and invasion. Further prognosis analysis indicated that high expression of *ZNF671* was associated with longer overall survival (OS) and that *ZNF671* expression is a favorable prognostic indicator in BRCA, CESC, HNSC, KIRP, LUAD, PAAD, SARC, and UCEC, but the mechanism remains unknown. Further studies are needed to clarify this issue. Our results confirm that *ZNF671* can be used as a biomarker, with low expression predicting worse outcomes in patients with BRCA, CESC, HNSC, KIRP, LUAD, PAAD, SARC, and UCEC.

## Author Contributions

JiZ and ZZ designed the research. JlZ, TX, YT, RL, BW, JL, and AX acquired and analyzed the data. JiZ, XH and YY wrote the manuscript.

### Conflict of Interest Statement

The authors declare that the research was conducted in the absence of any commercial or financial relationships that could be construed as a potential conflict of interest.
