# Corrigendum: Epigenetic-mediated downregulation of zinc finger protein 671 (*ZNF671*) predicts poor prognosis in multiple solid tumors

**DOI:** 10.3389/fonc.2023.1334056

**Published:** 2023-11-20

**Authors:** Jian Zhang, Ziqi Zheng, Jieling Zheng, Tao Xie, Yunhong Tian, Rong Li, Baiyao Wang, Jie Lin, Anan Xu, Xiaoting Huang, Yawei Yuan

**Affiliations:** ^1^ Department of Radiation Oncology, Affiliated Cancer Hospital and Institute of Guangzhou Medical University, Guangzhou, China; ^2^ State Key Laboratory of Respiratory Diseases, Guangzhou Institute of Respiratory Disease, Affiliated Cancer Hospital and Institute of Guangzhou Medical University, Guangzhou, China; ^3^ State Key Laboratory of Oncology in South China, Collaborative Innovation Center of Cancer Medicine, Sun Yat-sen University Cancer Center, Guangzhou, China; ^4^ Department of Radiation Oncology, Nanfang Hospital, Southern Medical University, Guangzhou, China

**Keywords:** epigenetic, *ZNF671*, prognosis, solid tumor, data mining

In the published article, there was an error in **Figure 6** as published. Due to incorrect use of images, we found a minor error in the HOS-ZNF671 (D). We repeated the experiments and corrected the results. The corrected **Figure 6** and its caption appear below.

**Figure 6:**
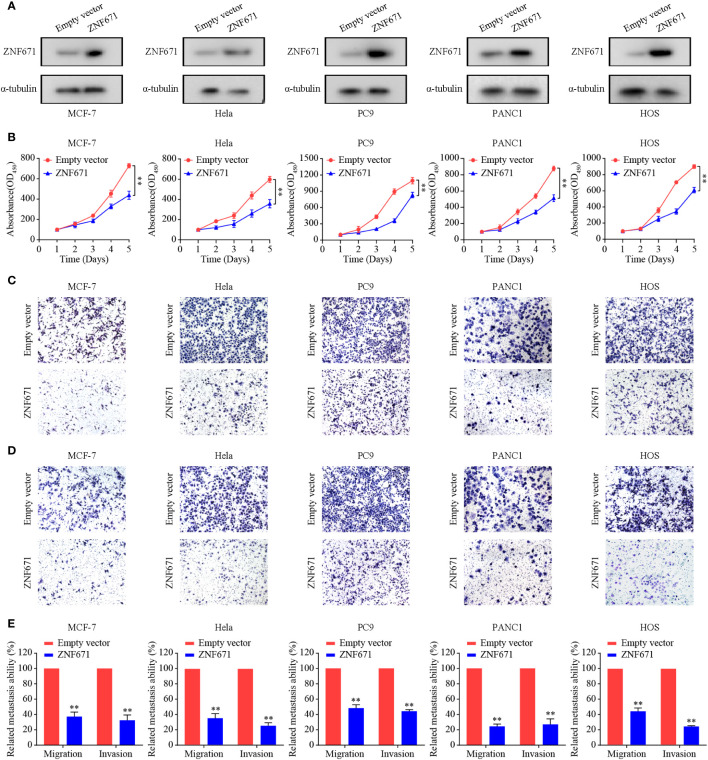
Effects of ZNF671 overexpression on cell proliferation, migration, and invasion *in vitro*. **(A)** Representative Western blot analysis of ZNF671 overexpression in MCF7, Hela, PC9, PANC1, and HOS cell lines. **(B)** The CCK-8 assay showed overexpression of ZNF671 reduced the viability of MCF7, Hela, PC9, PANC1, and HOS cells. **(C, D)** Representative images of the effects of ZNF671 overexpression on migratory and invasive abilities of cells as determined by Transwell migration **(C)** and invasion **(D)** assays. **(E)** Quantification of the effects of ZNF671 overexpression on migratory and invasive abilities. All of the experiments were performed at least three times. Data presented are the mean ± SD; **P < 0.01 compared with control using Student’s *t*-test.

The authors apologize for this error and state that this does not change the scientific conclusions of the article in any way. The original article has been updated.

